# Soluble urokinase plasminogen activator receptor levels are predictive of COVID-19 severity in Afro-Caribbean patients

**DOI:** 10.2217/bmm-2021-0669

**Published:** 2022-01-27

**Authors:** Mael Padelli, Papa Gueye, Diane Guilloux, Rishika Banydeen, Valentine Campana, Andre Cabie, Remi Neviere

**Affiliations:** ^1^University Hospital of Martinique, Department of Biochemistry, Fort-de-France, 97261, Martinique, France; ^2^University Hospital of Martinique, Department of Emergency Medicine, Fort-de-France, 97261, Martinique, France; ^3^University Hospital of Martinique, Department of Critical Care Medicine, Fort-de-France, 97261, Martinique, France; ^4^University Hospital of Martinique, Department of Epidemiology & Biostatistics, Fort de France, 97261, Martinique, France; ^5^Centre d’Investigation Clinique Antilles Guyane, Inserm CIC 1424, Fort de France, 97261, Martinique, France; ^6^University Hospital of Martinique, Department of Infectious Diseases, Fort-de-France, 97261, Martinique, France; ^7^University Hospital of Martinique, Department of Cardiology, Fort de France, 97261, Martinique, France; ^8^Université des Antilles, Cardiovascular Research Team EA7525, Fort de France, 97261, France

**Keywords:** Afro-Caribbean, chest CT severity score, COVID-19, intensive care, National Early Warning Score, soluble urokinase plasminogen activator receptor

## Abstract

**Aim:** To investigate association between soluble urokinase plasminogen activator receptor (suPAR) plasma levels at admission and incidence of complications in COVID-19 patients. **Patients & methods:** We considered Afro-Caribbean patients (n = 64) admitted to the hospital between 1 February 2020 and 28 February 2021. Primary outcome was time from the hospital admission until intensive care unit care or death. **Results:** Primary outcome (hazard ratio, HR [95%CI]) was associated with higher CT scan severity score (3.18 [1.15–8.78], p = 0.025), National Early Warning Score (NEWS2; 1.43 [1.02–2.02], p = 0.041) and suPAR (1.28 [1.06–2.06], p = 0.041). Kaplan–Meier analysis indicated patients with suPAR level above 8.95 ng/ml had a worse outcome (7.95 [3.33–18.97], p < 0.001). **Conclusion:** Our study suggests that COVID-19 patients with increased baseline suPAR levels are at a high risk of complications.

## Lay abstract

Our aim was to investigate association between the plasma levels of soluble urokinase plasminogen activator receptor (suPAR) at admission and incidence of complications in COVID-19 patients. Increased suPAR level has been previously associated with activation of inflammation and coagulation, which important features of COVID-19. We considered Afro-Caribbean patients admitted to the hospital between 1 February 2020 and 28 February 2021. Primary outcome was time from the hospital admission until intensive care unit care or death. The use of an integrative prediction tool which combines simple clinical score (NEWS2), imaging technique (chest CT severity score) and suPAR plasma levels has potent predictive value for COVID-19 outcome.

COVID-19 is related to a novel coronavirus, the SARS-CoV-2 [1]. Most patients with COVID-19 present with mild to moderate symptoms, but 5–10% of cases develop pneumonia requiring prolonged hospitalization and occasionally ICU management for acute respiratory distress syndrome (ARDS) [1–3]. In addition, well-defined clinical risk factors [2,4,5], identification of blood biomarkers that might predict patient’s deterioration represent a critical issue at the hospital admission [4–6]. Interestingly, when combined to routine clinical score (e.g., NEWS2 score), biomarkers such as soluble urokinase plasminogen activator receptor (suPAR) and IL-6 may be invaluable when triaging patients on the hospital admission to detect who can be safely discharged versus those who need careful clinical monitoring [7].

suPAR is a soluble receptor released from proteolytic cleavage of uPAR receptor shedding at the surface of blood mononuclear and endothelial cells along with the activation of inflammatory processes [8]. It has been suggested that suPAR is a proinflammatory biomarker that can be used as an early mortality indicator in these diseases [8,9]. Due to the urokinase receptor system is a regulator at the crossroad of inflammatory, immune, coagulation and fibrinolytic responses, determination of suPAR levels may be well suitable in the diagnosis, assessment and prognosis of COVID-19. Clinical presentation and predictors of outcome in the African descents hospitalized with COVID-19 have not been extensively investigated despite the disproportionately higher burden and mortality compared with other ethnic groups [10,11]. High incidence of co-morbidities and carriage of genetic variants of inflammatory cytokine and coagulation factors may explain why African populations are badly hit by the COVID-19 pandemic [12,13].

Whether prognostic biomarkers of COVID-19 severity evaluated in Caucasians on the hospital admission would be invaluable when triaging African descent patients is largely unknown. Our objective was to report clinical features and main predictor factors of disease severity at the time of admission in Afro-Caribbean COVID-19 patients hospitalized in Martinique, an outermost French Caribbean Island. We prospectively conducted a cohort study to investigate association between plasma levels of suPAR at admission and the incidence of severe complications in hospitalized COVID-19 patients.

## Methods

### Patients & data collection

An ethnic group of Afro-Caribbean with total or partial ancestry from any of the black racial groups of Africa was recruited via EPIC, an observational COVID-19 study promoted by the University Hospital of Martinique, under the authority of our General Data Protection Regulation controller. The EPIC study was conducted in accordance with the amended Declaration of Helsinki (http://www.wma.net/en/30publications/10policies/b3/). Written informed consent was obtained from all patients. The EPIC protocol was approved by the local Institutional Review Board of the University Hospital of Martinique (reference number: 2020/048).

Only patients greater than 18 years of age with a positive PCR result for SARS-CoV-2 were included. Medical records of COVID-19 patients were collected between 1 February 2020 and 28 February 2021, based on the earliest available clinical and biochemical information. All patients were hospitalized in the emergency room (ER) of the University Hospital of Martinique. All age ranges were included. Exclusion criterion was inability or refusal to consent, pregnancy, uncertain African descent, incomplete medical record and absence of biological evaluation at admission (including suPAR level determination). Due to the greater risk of mortality and critical care admission in adults with nosocomial COVID-19, in-hospital COVID-19 was considered as exclusion criteria in our study. Information recorded included medical history, exposure history, co-morbidities, symptoms, laboratory findings and computed tomographic scans. Two expert chest radiologists retrospectively evaluated all CT scans on a picture archiving and communication system workstation. A radiological severity score of 0–4 was assigned to each lung depending on the extent of involvement by consolidation or ground glass opacities: 0 = no involvement, 1 = <25%, 2 = 25–49%, 3 = 50–75%, 4 = >75% involvement. A total score (numerical) was calculated by summing the five lo-be scores (range, 0–20) and a simplified (category) severity score ranging from mild (<7), moderate (8–17) and severe (18 or more) was used [14,15].

Routine biochemistry and hematology parameters as well as conventional biomarkers were prospectively measured on the first blood samples withdrawn at admission. For other potential predictive markers, such as IL-6, procalcitonin and suPAR, analysis was performed on frozen samples prepared with blood samples withdrawn at admission. The window of time between the measurement of suPAR and the measurement of routine markers was less than 3 h. Laboratory staff was unaware of clinical outcome and were therefore functionally blinded. Measurements suPAR were performed by an enzyme immunoassay method (suPARnostic™, ViroGates, Lyngby, Denmark) using a Cobas c501 analyzer (Roche Diagnostics, Mannheim, Germany). Procalcitonin values were measured using an enzyme-linked fluorescent immunoassay (BioMerieux, Marcy-l’Étoile, France) on a Cobas e601 analyzer (Roche, Basel, Switzerland). IL-6 measurements were performed by an electrochemiluminescence method (Roche Diagnostics) using a Cobas e601 analyzer (Roche). Measurements of procalcitonin, IL-6 and suPAR were performed and reported by one technician who was blinded to information.

### Outcomes

The primary outcome of prediction was a composite outcome of ICU admission requiring noninvasive or mechanical ventilation or death.

### Statistics

Descriptive statistics were used to summarize baseline demographic characteristics, clinical characteristics, laboratory results and clinical outcomes. Continuous variables were presented as mean and standard deviation (SD) or median and interquartile range (IQR) as appropriate. The Kolmogorov–Smirnov test was conducted to test the normality of distribution. Categorical variables were presented as percentage. Between-group differences were assessed using *t*-test and Chi-square for normally distributed data and dichotomized data, respectively. The Mann–Whitney U test was used for non-normally distributed data comparisons [16]. Receiver-operating curve (ROC) analysis was used to establish cutoffs of relevant variables and their prognostic value and to calculate their area under the curves (AUC). Survival was evaluated with Cox proportional hazards regression analysis, providing estimated hazard ratios (HR) and Kaplan–Meier curves. The variables were first explored with univariate Cox regression analysis. For multivariate regression, a backward conditional model was used with stepwise entry and removal criteria set at 0.05 and 0.10, respectively. In the multivariate analysis, variables were entered as continuous variables and the entry procedure was used to determine independent predictors. Goodness-of-fit statistic test for the Cox proportional hazards model which is of the Hosmer and Lemeshow type was used. Because of the relatively small number of clinical events, we restrained the number of variables in multivariate Cox models, in accordance with results of the univariate Cox analysis as well as clinical relevance of variables [17]. A significance level of 0.05 was chosen for all tests. Statistical analyses were performed using the Statistical Package for the Social Sciences (SPSS, Inc., IL, USA).

## Results

**Figure 1.**
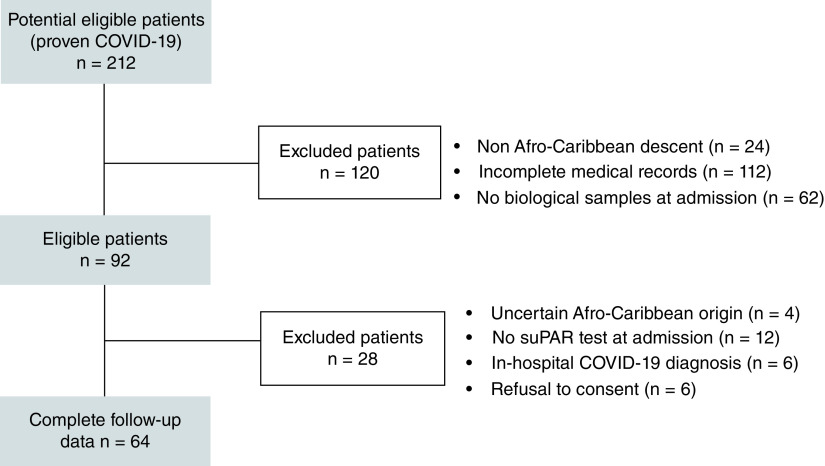
Study flowchart.

**Figure 2.**
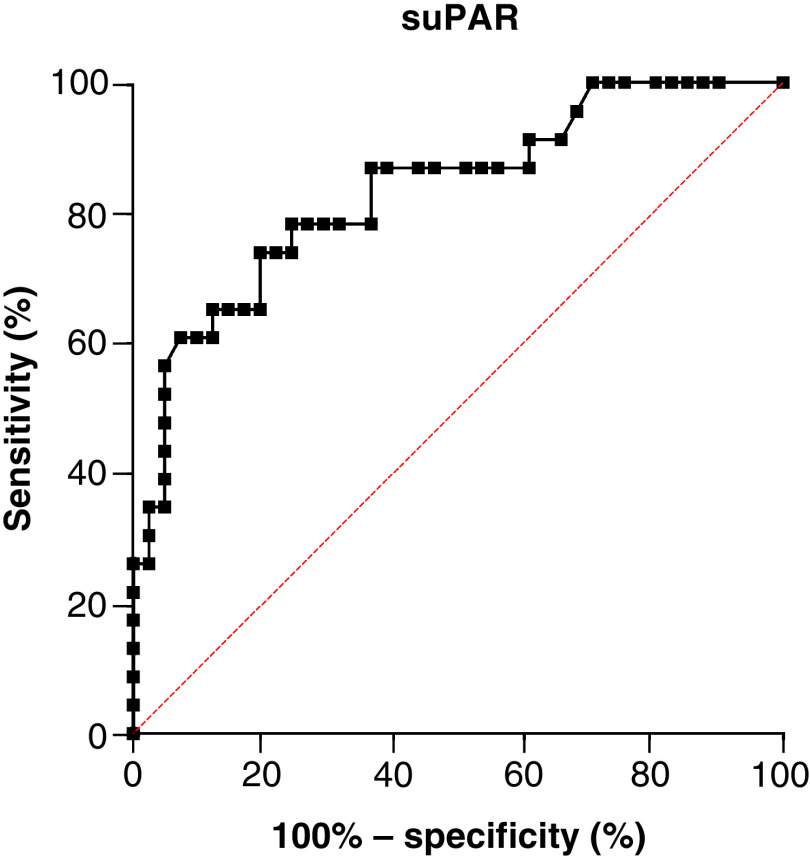
Receiver-operating characteristic analysis for the prediction of outcome (intensive care unit admission or death) (soluble urokinase plasminogen activator receptor). ROC-derived cutoff for plasma suPAR concentration was 8.95 ng/ml (AUC: 0.834, sensibility 78%, specificity 76%). AUC: Area under the curve; ROC: Receiver-operating characteristic; suPAR: Soluble urokinase plasminogen activator receptor.

**Figure 3.**
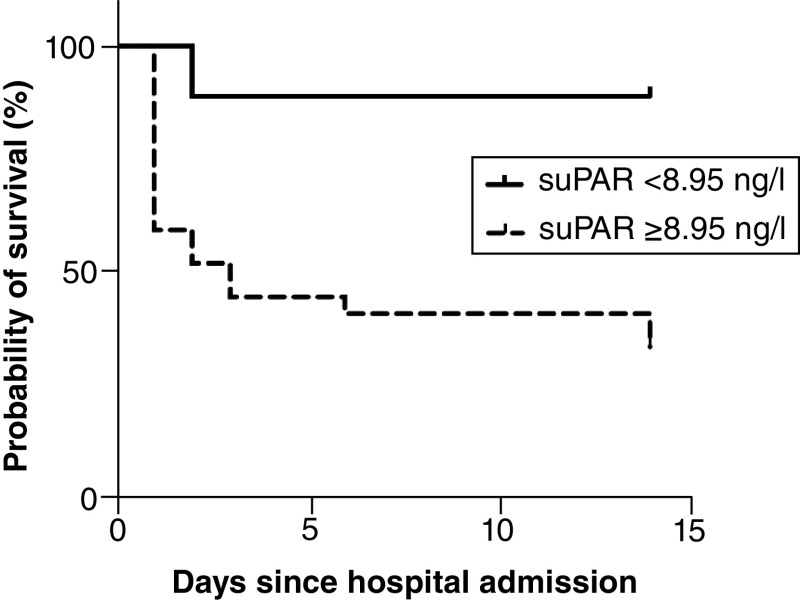
Kaplan–Meier survival curves from the Cox model for primary outcome (composite of intensive care unit admission or death) for plasma-soluble urokinase plasminogen activator receptor levels. A log-rank test stratified by group indicated a significant difference in the survival curves (HR: 7.95 [95% CI: 3.33–18.97]; p < 0.001). HR: Hazard ratio.

**Figure 4.**
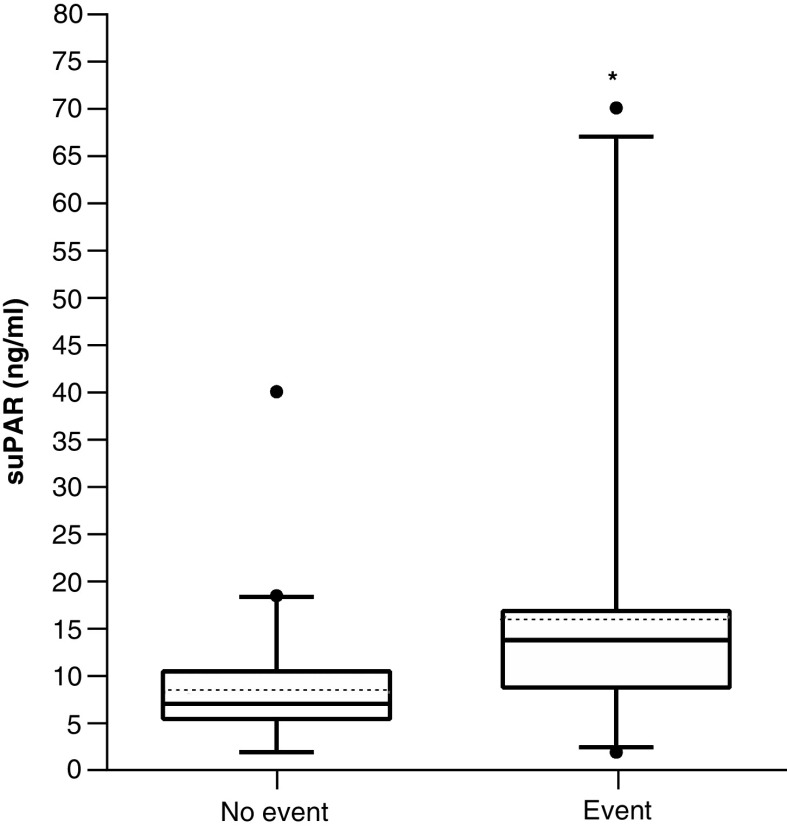
Box plot of plasma-soluble urokinase plasminogen activator receptor levels (ng/ml) in COVID patients with and without primary outcome defined as intensive care unit admission requiring noninvasive or mechanical ventilation or death. *Indicates p < 0.05 (the Mann–Whitney U test). Median (plain line) and mean (dashed line) are displayed.

Out of the 212 Afro-Caribbean patients hospitalized at the Martinique’ University Hospital during the study period (1 February 2020–28 February 2021), only patients with RT-PCR-confirmed COVID-19 and complete medical and biological record were included. Out of the 92 screened patients, only 64 were eventually included in our study (Figure 1). Reasons for exclusion were: uncertain Afro-Caribbean origin, absence of suPAR test performed at the time of admission in the emergency department, in-hospital COVID-19 diagnosis and inability or refusal to consent. We had no loss to follow-up. Table 1 shows the demographics of the cohort. All participants had African ancestries. Mean age of patients was 68 years, 67% were male. Hypertension (58%), obesity (47%) and diabetes (38%) were the most common co-morbidities. Out of the 64 COVID-19 patients, 23 (n = 23) had the primary composite outcome (ICU admission requiring noninvasive or mechanical ventilation and death). Compared with hospitalized patients who survived or did not required ICU management, patients who experienced the primary outcome had higher radiological severity score on CT scan (median of 3, IQR [3–4] vs median of 2, IQR [2–3]; p < 0.001) and higher National Early Warning Score (NEWS2) at admission (median of 4, IQR [3–5] vs median of 3, IQR [2–3]; p = 0.002). Admission blood tests are recorded in Table 2. Cox proportional hazards regression analysis indicated that chest CT scan severity score (3.18 [1.15–8.78], p = 0.025), NEWS2 (1.43 [1.02–2.02], p = 0.041) and suPAR (1.28 [1.06–2.06], p = 0.041) were found independent risks factor for the incidence of events (i.e., ICU admission or death) during follow-up (Table 3). The ROC curve and the AUC with 95% CI were used to assess the ability of suPAR plasma levels in distinguishing patients requiring or not ICU admission (Figure 2). The best values of NEWS2 and of plasma suPAR concentration to predict outcome were, respectively, a NEWS2 score of 3 (AUC: 0.740 [95% CI: 0.612–0.867], sensitivity 61%, specificity 80%) and suPAR plasma level of 8.95 ng/ml (AUC: 0.834 [95% CI: 0.729–0.940], sensibility 78%, specificity 76%). Figure 3 shows survival curves for the composite end point with suPAR. Comparison of suPAR plasma levels between patients requiring or not ICU admission is displayed Figure 4.

**Table 1.** Characteristics of Afro-Caribbean COVID-19 patients at the hospital admission according to primary outcome.

Parameters	All patients (n = 64)	No (n = 41)	Yes (n = 23)	p-value^†^
Age, years	68 ± 12	68 ± 13	69 ± 10	0.632
Male gender, n (%)	43 (67)	32 (67)	11 (69)	0.568
BMI, kg/m^2^	29 ± 9	28 ± 9	30 ± 8	0.528
**Medical history**				
Diabetes, n (%)	24 (38)	6 (38)	18 (38)	0.621
Hypertension, n (%)	37 (58)	10 (63)	27 (56)	0.445
Cardiovascular disease, n (%)	6 (9)	4 (8)	2 (13)	0.471
Chronic kidney disease, n (%)	6 (9)	5 (11)	6 (9)	0.529
Asthma, n (%)	5 (8)	3 (6)	2 (13)	0.367
Cancer, n (%)	5 (8)	5 (10)	0 (0)	0.225
Temperature, °C	37.7 ± 0.9	37.5 ± 0.7	38 ± 1.1	0.02
NEWS2 score	3 (2–4)	3 (2–3)	4 (3–5)	0.002
NEWS2 score >3	22 (34)	8 (20)	14 (61)	<0.001
Systolic blood pressure, mmHg	129 ± 19	130 ± 16	128 ± 23	0.679
Diastolic blood pressure, mmHg	75 ± 11	77 ± 12	72 ± 8	0.133
Heart rate, min^-1^	84 ± 17	84 ± 11	83 ± 25	0.902
Respiratory rate, min^-1^	26 ± 8	24 ± 6	31 ± 9	0.001
Pulse oximetry (%)	95 (93–97)	95 (94–97)	92 (90–95)	<0.001
Glasgow score	15 ± 2	15 ± 1	14 ± 3	0.015
Grade pulse oximetry SpO_2_, n (%)				<0.001
– <= 93%	24 (38)	8 (20)	16 (70)	
– 94–96%	22 (34)	20 (49)	2 (9)	
– >96%	18 (28)	13 (31)	5 (21)	
Chest CT severity score, n (%)				<0.001
– Mild	34 (53)	30 (73)	3 (13)	
– Moderate	21 (33)	10 (27)	11 (48)	
– Severe	9 (14)	0 (0)	9 (39)	

Primary outcome includes the development of acute respiratory distress syndrome, intensive care unit admission or death from any cause. See *Methods* section for definition Chest CT severity score.

† Results are presented as mean ± standard deviation or median and IQRs (25–75%). Statistical significance was set at p < 0.05.

CT: Computerized tomography; IQR: Interquartile range; NEWS: National Early Warning Score; SpO_2_: Pulse oximetry.

**Table 2.** Laboratory features at admission of Afro-Caribbean COVID-19 patients according to primary outcome.

Parameters	All patients (n = 64)	Primary outcome		p-value
		No (n = 41)	Yes (n = 23)	
C-reactive protein (mg/l)	119 (56–221)	79 (36–128)	206 (145–278)	<0.001
Procalcitonin (ng/ml)	0.2 (0.1–0.4)	0.1 (0.1–0.2)	0.3 (0.2–0.7)	0.003
IL-6 (pg/ml)	54 (23–112)	37 (18–58)	114 (55–205)	0.001
suPAR (ng/ml)	8.0 (6.0–13.8)	7.1 (4.9–9.1)	14.1 (9.1–17.7)	<0.001
Neutrophils (10^9^/l)	4.8 (3.4–6.6)	4.3 (2.9–5.2)	7.0 (4.5–8.5)	<0.001
Lymphocytes (10^9^/l)	1.1 (0.7–1.6)	1.2 (0.8–1.6)	0.9 (0.6–1.4)	0.295
Platelets (10^9^/l)	224 (163–293)	222 (168–292)	231 (148–298)	0.073
Prothrombin time (%)	76 (68–83)	79 (73–88)	71 (67–80)	0.014
Fibrinogen (g/l)	5.8 (4.7–6.7)	4.9 (4.5–6.5)	6.2 (4.9–7.2)	0.029
D-dimer (μg/ml)	0.9 (0.5–1.7)	0.8 (0.5–1.1)	1.3 (0.6–2.4)	0.520
Vitamin D (nmol/l)	26 (19–32)	37 (20–32)	26 (17–31)	0.385
Iron (μmol/l)	5.7 (3.9–8.5)	6.1 (4.1–8.9)	4.9 (3.2–7.6)	0.154
Transferrin (mg/l)	1.5 (1.3–1.8)	1.6 (1.5–1.9)	1.5 (1.3–1.6)	0.039
Creatinine (μmol/l)	94 (71–116)	94 (73–118)	93 (68–116)	0.810
Bilirubin (μmol/l)	9.5 (6.8–13.8)	9.8 (6.7–12.3)	9.5 (6.8–15.4)	0.343
ASAT (unit/l)	52 (32–75)	49 (29–67)	68 (41–113)	0.099
ALAT (unit/l)	37 (17–57)	30 (16–52)	46 (28–74)	0.269
LDH (unit/l)	365 (273–471)	328 (243–468)	402 (347–621)	0.042
Glucose (mmol/l)	6.6 (6.0–8.9)	6.5 (5.7–7.8)	7.0 (6.2–9.4)	0.499
Albumin (g/l)	27 (24–31)	28 (26–32)	24 (21–30)	0.501
CK (unit/l)	259 (106–603)	198 (99–418)	309 (110–1828)	0.134
Troponin (ng/l)	11 (10–27)	10 (10–17)	19 (10–37)	0.046

Primary outcome includes the development of acute respiratory distress syndrome, intensive care unit admission or death from any cause. Results are presented as median (IQR).

Between group differences are assessed using Mann–Whitney U test for non-normally distributed data. Statistical significance was set at p < 0.05.

ALAT: Alanine transaminase; ASAT: Aspartate aminotransferase; CK: Creatine kinase; IQR: Interquartile range; LDH: Lactate dehydrogenase.

**Table 3.** Predictors of intensive care unit hospitalization or death in Afro-Caribbean COVID-19 patients: univariate and multivariate COX analyses.

Parameters	Univariate COX analysis		Multivariate COX analysis	
	HR (95% CI)	p-value	HR (95% CI)	p-value
CT scan severity score	5.85 (2.10–16.29)	0.001	3.18 (1.15–8.78)	0.025^†^
NEWS2	1.54 (1.12–2.12)	0.008	1.43 (1.02–2.02)	0.041^†^
Temperature	1.54 (0.99–2.39)	0.054		
Respiratory rate	1.09 (1.03–1.14)	0.002		
Glasgow score	0.80 (0.69–0.93)	0.004		
Pulse oximetry	0.79 (0.69–0.91)	0.001		
C-reactive protein	1.01 (1.00–1.02)	<0.001		
Procalcitonin	1.27 (1.05–1.55)	0.017		
suPAR	1.06 (1.2–1.07)	<0.001	1.28 (1.06–2.06)	0.041^†^
Neutrophils	1.31 (1.86–1.45)	<0.001		
Prothrombin time	0.97 (0.95–0.99)	0.028		

† Variables with significant association in univariate COX regression analysis (p < 0.15) were considered for multivariate COX analysis. Variables entered into the initial multivariate model for composite primary outcome: CT scan severity score, NEWS2, C-reactive protein, neutrophils, procalcitonin, prothrombin time and suPAR. Statistical significance level for multivariate analysis set at p < 0.05. Goodness-of-fit of final multivariate model: p = 0.580 (the Hosmer–Lemeshow test).

CT: Computerized tomography; LDH: Lactate dehydrogenase; NEWS2: National Early Warning Score; suPAR: Soluble urokinase plasminogen activator receptor.

## Discussion

Consistent with previous studies performed in patients with various levels of COVID-19 illness [7,14,18–21], we found that clinical and laboratory parameters previously reported to have prognostic significance in COVID-19 patients may be used to predict clinical deterioration in Afro-Caribbean people. Compared with hospitalized patients who survived or did not required ICU management, COVID-19 patients who experienced the primary outcome (i.e., need for ICU management or death during follow-up) had higher chest CT scan severity score, NEWS2, and suPAR plasma levels at admission, which were found independent risks factor for the incidence of events.

Use of the NEWS2 has been previously shown to help manage medical emergencies [22]. The NEWS that is widely used in the healthcare setting both in the UK and abroad, can reliably detect deterioration in adults, trigger medical review, treatment and escalation of care [23,24). During coronavirus outbreaks, NEWS2 score has been successful use to rapidly identify COVID-19-infected patients with high risk of clinical deterioration, thus indicating whenever a patient may need early hospitalization, critical care or can be treated at home by their general practitioner. Consistently with previous studies, NEWS2 score had an AUC of 0.740 in our cohort. Our study performed in Afro-Caribbean patients at the University Hospital of Martinique confirm that calculation of the NEWS2 at the time of hospital admission NEWS2 has good predictive ability in patients with COVID-19. Interestingly, our study confirms that chest CT scan plays an important role in severity stratification and may be considered an important tool to predict the outcome (ICU admission or death) of COVID-19 patients [25].

Previous studies have emphasized that suPAR elevation correlates with clinical severity and can play a critical role in the development of pulmonary, renal and cardiac complications in COVID-19 patients [7,14,18–21]. In the present study, we tested whether increased plasma levels of suPAR measured at admission would predict severe COVID-19 complications in hospitalized patients. We consistently found that measurement of suPAR levels at admission of COVID-19 patients with African descent represents a valuable tool for helping risk stratification accuracy and can predict the risk of developing severe consequences of SARS-CoV-2 infection. Both incidence of the composite event and cut-off point derived for suPAR (8.95 ng/ml) were found quite high in comparison with previous studies [7,14,20,21]. We attributed this difference to the fact that patients attending the emergency room of the University Hospital of Martinique had more severe COVID-19 status, which is suggested by clinical and biological abnormal presentation at the hospital admission.

It is well accepted that a reliable prediction tool should integrate simple clinical score, imaging techniques as well as biological features. We found that NEWS2, chest CT severity score and suPAR plasma levels at admission have potent predictive value for COVID-19 outcome. Among fast biomarkers for risk stratification in COVID-19 patients, suPAR not only reflect specific biological conditions (i.e., endothelial activation, inflammation, as well as hypofibrinolytic condition and hypercoagulable state) but also is influenced by pre-existing co-morbidities. In Afro-Caribbean patients, these original features may be of critical importance due to the carriage of genetic variants of inflammatory cytokine and coagulation factors, along with co-morbidities such as cardiovascular diseases, diabetes, chronic lung disease and obesity, which may largely explain why African populations are badly hit by the COVID-19 pandemic [12,13].

### Strength & limitation of the study

The major limitation of this single-center study is the limited sample size, which may have led to imprecise estimates of biomarker performance. However, our results may be of sufficient practical significance because our study represents a unique experience of COVID-19 patient management in a small oversea island, which can offer only one ICU facilities. These specific geographic and healthcare conditions result in the inclusion of a homogeneous cluster of COVID-19 patients who were evaluated and treated the same way.

Our findings have been generated in a specific Afro-Caribbean population living in Martinique, a French Caribbean island. Whereas similar prognostic indicators could be identified in other ethnic groups is mandatory, it might be difficult in Martinique because Afro-descendant communities represent approximately 67% of the population in the Caribbean countries and more than 90% of patients presenting at the Emergency Room had African ancestries.

## Clinical significance

Because some patients with confirmed COVID-19 develop rapidly progressive respiratory failure and need for ICU management, identification of blood biomarkers that might predict patient’s deterioration represents a critical issue at the hospital admission.

We found that measurement of suPAR levels at admission of COVID-19 patients with African descent represents a valuable tool for helping risk stratification accuracy and can predict the risk of developing severe consequences of SARS-CoV-2 infection.

## Conclusion

suPAR is a potential biomarker for triage of patients with COVID-19 symptoms at admission in the emergency room. Our study’s results demonstrate that COVID-19 patients with increased baseline suPAR levels are at a high risk of developing complications, which can significantly improve COVID-19 triage. Future studies might clearly be considered integrating plasma suPAR and other clinical, radiological and laboratory biomarkers into prognostic models to improve risk prediction accuracy in COVID-19 patients.

**Summary points**

- Well-defined clinical risk factors predicting COVID-19 patient’s deterioration have been proposed.
- Biomarkers that predict the clinical course of COVID-19 would be invaluable at the time of hospital admission.
- Several blood-based biomarkers have been suggested in Caucasian people but have not been tested in a prospective Afro-Caribbean cohort.
- In this prospective Afro-Caribbean cohort, chest computerized tomography severity score and National Early Warning Score were found independent risks factor for the incidence of events (i.e., intensive care unit admission or death) during follow-up.
- Increased soluble urokinase plasminogen activator receptor level was associated with activation of inflammation and coagulation, which are important features of COVID-19.
- Level of soluble urokinase plasminogen activator receptor plasma at admission has potent predictive value for COVID-19 outcome in Afro-Caribbean patients.
